# Evidence That Agouti-Related Peptide May Directly Regulate Kisspeptin Neurons in Male Sheep

**DOI:** 10.3390/metabo11030138

**Published:** 2021-02-26

**Authors:** Christina M. Merkley, Sydney L. Shuping, Jeffrey R. Sommer, Casey C Nestor

**Affiliations:** Department of Animal Science, North Carolina State University, Raleigh, NC 27695, USA; cmmerkle@ncsu.edu (C.M.M.); slshupin@ncsu.edu (S.L.S.); jrsommer@ncsu.edu (J.R.S.)

**Keywords:** kisspeptin, AgRP, sheep, reproduction, LH

## Abstract

Agouti-related peptide (AgRP) neurons, which relay information from peripheral metabolic signals, may constitute a key central regulator of reproduction. Given that AgRP inhibits luteinizing hormone (LH) secretion and that nutritional suppression of LH elicits an increase in AgRP while suppressing kisspeptin expression in the arcuate nucleus (ARC) of the hypothalamus, we sought to examine the degree to which AgRP could directly regulate ARC kisspeptin neurons. Hypothalamic tissue was collected from four castrated male sheep (10 months of age) and processed for the detection of protein (AgRP input to kisspeptin neurons) using immunohistochemistry and mRNA for melanocortin 3 and 4 receptors (MC3R; MC4R) in kisspeptin neurons using RNAscope. Immunohistochemical analysis revealed that the majority of ARC kisspeptin neurons are contacted by presumptive AgRP terminals. RNAscope analysis revealed that nearly two thirds of the ARC kisspeptin neurons express mRNA for MC3R, while a small percentage (<10%) colocalize MC4R. Taken together, this data provides neuroanatomical evidence for a direct link between orexigenic AgRP neurons and reproductively critical kisspeptin neurons in the sheep, and builds upon our current understanding of the central link between energy balance and reproduction.

## 1. Introduction

Proper nutritional balance is paramount to the capacity for reproduction in mammals. Favorable metabolic conditions allow for pubertal development and fertility, while an unfavorable metabolic state such as undernutrition, can suppress reproductive function. This, at least in part, occurs via central integration of metabolic signals given that negative energy balance inhibits gonadotropin-releasing hormone (GnRH), and subsequently luteinizing hormone (LH), release [1,2,3]. Although GnRH neurons serve as the final common conduit from the central nervous system controlling reproduction, they appear to lack the appropriate receptors to directly respond to a metabolic hormone such as leptin [4,5,6]. Thus, changes in peripheral metabolic signals reflective of undernutrition (i.e., lower circulating leptin concentrations) must recruit inhibitory afferents and/or block stimulatory inputs to GnRH neurons in order to ultimately suppress GnRH/LH release during a negative energy state. One such stimulatory afferent is the neuropeptide kisspeptin, which has perikarya located primarily in two areas of the ventral forebrain, the anterior ventral periventricular area (AVPV; rodents)/preoptic area (POA; non-rodents) and the arcuate nucleus (ARC) of the hypothalamus [7,8,9]. Indeed, the stimulatory action of kisspeptin on LH secretion has been confirmed in several species [10], and the vast majority of GnRH neurons colocalize Kiss1R [11,12,13,14], supporting the idea of a direct stimulatory action of kisspeptin on GnRH neurons. Arcuate kisspeptin neurons in particular are identified as playing a dominant role in pulsatile GnRH/LH release [15,16], and anatomical evidence has shown that up to 60% of GnRH cell bodies receive kisspeptin input arising from the ARC kisspeptin population [17]. Indeed, food restriction has been shown to reduce expression of ARC kisspeptin [18,19,20,21,22]. Furthermore, there is evidence to show that kisspeptin neurons express leptin receptors [23,24,25], but others have failed to demonstrate activation of leptin signaling in these neurons in vivo [26]. Thus, central regulation of GnRH/LH secretion during undernutrition likely incorporates other afferent inputs to ARC kisspeptin neurons.

Known for an orexigenic role in energy homeostasis, neurons that express agouti-related peptide (AgRP) may also play an important role to relay metabolic signals to reproductive axis. Located in the ARC, AgRP neurons express leptin receptors [27], and delayed puberty onset in leptin receptor-deficient mice can be rescued with select expression of leptin receptor within AgRP neurons [28]. With data demonstrating that central administration of AgRP inhibits LH secretion [29] and that undernutrition increases AgRP expression [30,31,32], AgRP signaling may represent a means whereby undernutrition inhibits GnRH/LH secretion. Interestingly, nearly all ARC AgRP neurons co-express neuropeptide Y (NPY) [32], and reciprocal connections between NPY neurons and kisspeptin cells have been reported [24]. However, given that other populations of NPY perikarya exist outside of the ARC [33], the degree to which AgRP neurons innervate ARC kisspeptin neurons is unclear, and remains to be examined. Furthermore, although classically known as the endogenous antagonist to α-melanocyte stimulating hormone (αMSH), a neuropeptide produced by anorexigenic pro-opiomelanocortin (POMC) neurons, AgRP alone has been shown to inhibit cells that express melanocortin 3 receptors (MC3R) and melanocortin 4 receptors (MC4R) via G_i/o_ signaling [34,35]. Work in mice has revealed very few ARC kisspeptin neurons express MC4R [25], but our recent work in sheep [36] led us to investigate the potential for AgRP regulation of ARC kisspeptin neurons in the ovine model. In this study, we examine the degree to which ARC kisspeptin perikarya are innervated by presumptive AgRP terminals using dual-label immunofluorescence, and utilizing a relatively new fluorescent in situ hybridization technique, RNAscope, we characterize the degree to which arcuate kisspeptin neurons colocalize MC3R and MC4R.

## 2. Results

### 2.1. AgRP Inputs to Kisspeptin Cells

**Figure 1:**
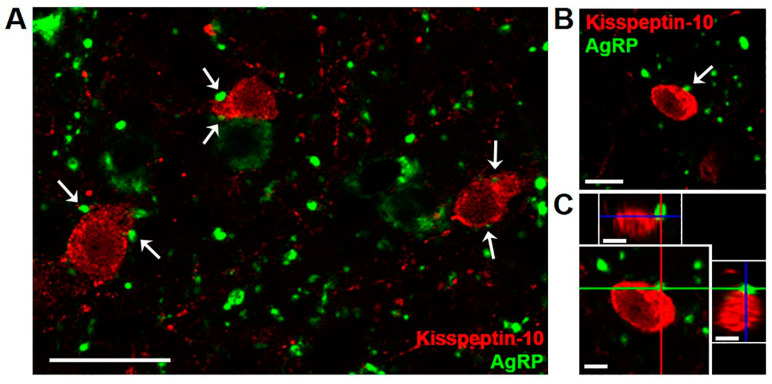
Confocal images (1.0 µm optical section; 40 × magnification) showing dual-label immunofluorescence for AgRP (green) and kisspeptin (red) in the arcuate nucleus of a castrated male sheep (wether). White arrows (**A**,**B**) indicate examples of AgRP-immunoreactive (ir) terminals in apposition to arcuate kisspeptin neurons, with AgRP-ir cell bodies and fibers seen in the vicinity. Orthogonal views (**C**) confirm close contact of an AgRP-labeled bouton to a kisspeptin cell body. Red, green, and blue lines in (**C**) indicate X, Y, and Z planes, respectively. Scale bars = 25 µm (**A**), 10 µm (**B**,**C**).

In ARC tissue from 10 month old castrated male sheep (wethers), AgRP-immunoreactive (ir) terminals were observed in frequent apposition to kisspeptin cell bodies and fibers (Figure 1). A total of 171 kisspeptin cells were analyzed for AgRP inputs in four animals, and the majority (72.31 ± 7.6%) of kisspeptin neurons showed at least one close contact by AgRP-ir terminals. A subset of kisspeptin neurons that showed at least one AgRP-ir input was examined further, and the total number of putative close contacts onto the cell body or proximal dendrite were analyzed from z-stack images (1.0 µm optical sections) taken throughout the extent of the cell. The results indicated that each kisspeptin perikaryon received multiple AgRP-ir close contacts (4.92 ± 1.0 contacts/cell). As reported previously in sheep [37], no colocalization of kisspeptin and AgRP in cell bodies or fibers was observed. There were, however, some instances of kisspeptin-ir fibers in apposition to AgRP perikarya (data not quantified).

### 2.2. Colocalization of Kisspeptin, MC3R, and MC4R

**Figure 2:**
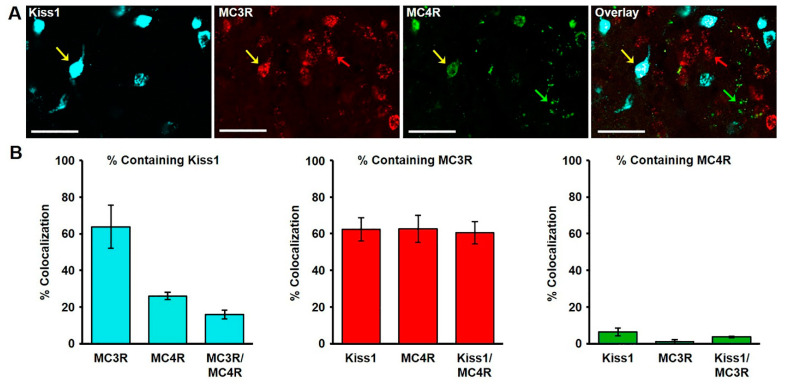
(**A**) Confocal image (1.0 µm optical section; 20 × magnification) of kisspeptin, MC3R, and MC4R mRNA-expressing cells in the arcuate nucleus (ARC) of a castrated male sheep. Yellow arrows indicate a Kiss1 cell expressing both MC3R and MC4R. Red and green arrows show MC3R and MC4R-expressing cells, respectively. Scale bar, 50 µm. (**B**) Mean (± SEM) percentage of kisspeptin (left), MC3R (middle), and MC4R (right) cells containing Kiss1, MC3R, and/or MC4R in the middle ARC.

The extent of colocalization of kisspeptin and the melanocortin receptors, MC3R and MC4R, was characterized in the ARC of wethers, and various instances of single, dual and triple mRNA-expressing cells were observed (Figure 2A). Of the MC3R mRNA-expressing cells (436 ± 30.4 examined per wether) in the middle ARC nearly two thirds (63.79 ± 11.7%; Figure 2B) colocalized mRNA for kisspeptin, while 14.42 ± 1.1% (Figure 2B) colocalized mRNA for MC4R. Of the total kisspeptin mRNA-expressing cells examined (431.75 ± 36.0 per wether), nearly two thirds co-expressed mRNA for MC3R (62.33 ± 6.3%), while only 6.33 ± 1.2% (Figure 2B) colocalize MC4R. Moreover, of the total MC4R mRNA-expressing cells identified (101.25 ± 5.3 per wether), 26.05 ± 2.1% contained kisspeptin (Figure 2B), while 62.59 ± 7.4% (Figure 2B) expressed mRNA for MC3R. Finally, we examined the degree to cells co-expressed in all three transcripts to find that 15.85 ± 2.4% of MC3R/MC4R cells express mRNA for kisspeptin, 60.53 ± 6.1% of Kiss1/MC4R cells express mRNA for MC3R, and 3.64 ± 0.4% of Kiss/MC3R cells express mRNA for MC4R (Figure 2B).

## 3. Discussion

In the present study, we provide neuroanatomical evidence to support the idea that AgRP signaling may constitute an important central regulatory mechanism in the control of reproduction. Through the use of immunohistochemical analysis, we have described that in the absence of gonadal sex steroids in male sheep, the majority of ARC kisspeptin neurons are innervated by AgRP terminals. In addition, using the fluorescent in situ hybridization technique, RNAscope, we have demonstrated that the majority of ARC kisspeptin neurons express MC3R, while a small percentage of ARC kisspeptin neurons express MC4R.

There is growing evidence that AgRP signaling plays a role in the regulation of reproduction, and the focus of this work was to investigate a central mechanism whereby AgRP could influence GnRH/LH secretion. Indeed, AgRP neurons have been shown to send axonal projections to brain regions where GnRH and kisspeptin neurons reside [38,39], but direct innervation of GnRH or kisspeptin neurons by AgRP has not been previously reported. The present finding showing the innervation of kisspeptin perikarya by AgRP terminals is in agreement with a previous report using optogenetics that demonstrated a functional connection between AgRP neurons and kisspeptin neurons in mice [40]. Given that nearly all ARC AgRP neurons co-express NPY [39] and that 15–30% of ARC kisspeptin neurons receive input from NPY cells in sheep [24], it is tempting to speculate that NPY inputs identified in contact with ARC kisspeptin neurons arise from AgRP neurons. However, several NPY-expressing neuronal populations exist in diencephalic regions outside of the ARC [33], and therefore we are unable to use NPY as an index of AgRP input. Since NPY and AgRP act through different receptors [41,42], NPY innervation of kisspeptin neurons should be viewed as an independent mechanism of regulation from that of AgRP. Moreover, herein we demonstrate that the majority of ARC kisspeptin neurons receive input from AgRP cells, and others have shown in sheep that 30–45% of ARC kisspeptin neurons receive input from POMC neurons [24]. Thus, we believe this strengthens the importance of melanocortin signaling for reproduction and warrants further investigation of the role each of these neuronal networks plays in the control of reproduction.

Central administration of AgRP has resulted in two in vivo effects on LH secretion, a reduction of LH secretion in ovariectomized, non-human primates [29], and a stimulation of LH secretion in gonadal-intact male rats [43]. While these divergent results could be due to differences in species, sex, and/or in sex steroid milieu, examination of the reproductive neurons that express melanocortin receptors and could directly mediate AgRP action becomes an important focus. In mice, over half of the GnRH neurons have been shown to express mRNA for MC4R [44], but AgRP alone has various effects on GnRH neurons in hypothalamic slice preparations (no effect [44], or stimulatory and inhibitory effects [45]). In addition, earlier work in mice reported that few kisspeptin neurons express MC4R [25]. However, based on our observations that ARC kisspeptin neurons express both MC3R and MC4R [36], we extended those findings to quantify the percentage of ARC kisspeptin neurons that express these receptors herein. In agreement with a relatively low percentage of kisspeptin neurons that express MC4R [25], we also observe a low co-expression of mRNA for kisspeptin and MC4R. However, when examining mRNA for MC3R, we found that a majority of ARC kisspeptin neurons express this receptor at a percentage that mirrors the percentage of ARC kisspeptin neurons which receive AgRP input. In agreement with our findings, more recent data in mice has revealed that ARC kisspeptin neurons express MC3R (personal communication with M. N. Bedenbaugh in the Simerly Lab). Given that AgRP is a potent endogenous antagonist of melanocortin receptors [46,47] and that central activation of melanocortin receptors stimulates LH secretion [48,49], we believe this new evidence provides strong support for the idea that AgRP can act directly at ARC kisspeptin neurons to influence reproduction.

The present findings in gonadectomized, male sheep lay important groundwork for future studies investigating central melanocortin signaling in the control of GnRH/LH release. While others report that there is no difference in POMC input to ARC kisspeptin neurons between gonad-intact and ovariectomized sheep [24], more work is needed to examine the impact of gonadal sex steroids on AgRP input to and/or melanocortin receptor expression in ARC kisspeptin cells of both sexes. Furthermore, now that we have a better understanding of the path AgRP has to influence key reproductive neurons, what remains to be determined is whether AgRP signaling acts to suppress kisspeptin neurons during undernutrition, a time in which AgRP [30,31,32] and MC4R [50] expression in the ARC is increased and αMSH expression is reduced [51]. Altogether, melanocortin signaling in ARC kisspeptin neurons may be a key mechanism whereby the status of low energy balance is relayed to the reproductive axis to ultimately reduce GnRH/LH secretion and impair reproduction at the level of the brain.

## 4. Materials and Methods

### 4.1. Animals

Four Suffolk wethers (male sheep castrated between four and six weeks of age) were approximately 10 months of age at the time of tissue collection in November. Prior to tissue collection, wethers were moved indoors for 14 days, housed individually, provided water ad libitum, and fed once daily (crude protein 12%, crude fat 2.5%, crude fiber 5.0%; Mule City Specialty Feeds, Bensen, NC, USA). Indoor lighting simulated natural day length. All procedures were approved by the North Carolina State University Animal Care and Use Committee (#17-020-B) and followed the National Institutes of Health guidelines for use of animals in research.

### 4.2. Tissue Collection

Tissue was collected as previously described [52]. Briefly, all wethers were heparinized (20,000 U, intravenous) and euthanized with an intravenous overdose of sodium pentobarbital (Euthasol; Patterson Veterinary, Greeley, CO, USA). Heads were removed and perfused via the carotid arteries with four liters of 4% paraformaldehyde (PFA) in 0.1 M phosphate buffer (PB; pH = 7.4) containing 0.1% sodium nitrite. Blocks of tissue containing the hypothalamus were removed and stored in 4% PFA for 24 h at 4 °C, then transferred to a 20% sucrose solution until sectioning. Frozen coronal sections (50 µm thickness) were cut using a freezing microtome into five parallel series and stored in cryopreservative solution until processing for immunofluorescence and RNAscope.

### 4.3. Dual-Label Immunofluorescent Detection of Kisspeptin and AgRP

To examine putative AgRP terminals in close contact with ARC kisspeptin cells, dual-label immunofluorescence was conducted for kisspeptin (Gift from Dr. I. Franceschini, Tours, France) [8,13,22,37,51] and AgRP (Antibodies Australia, Melbourne, Australia) [37,39]. Three sections in the middle ARC, defined as the level of the tubero-infundibular sulcus until the beginning of the formation of the mammillary recess of the third ventricle [53], were selected from a series of every fifth hypothalamic section (250 µm apart). Each coronal section selected for processing was cut at midline, with the left and right sides used for immunofluorescence and RNAscope (see below), respectively. All immunofluorescent procedures were performed on free-floating hemisections. On day one, sections were washed overnight in 0.1 M PB at 4 °C on a rocking shaker to remove excess cryoprotectant. All subsequent steps were conducted at room temperature (RT). On day two, sections were washed four times (5 min each) in 0.1 M phosphate buffered saline (PBS; pH = 7.4), then placed into 10% H_2_O_2_ (diluted in 0.1 M PBS; 10 min), followed by four washes (5 min each) in PBS. Tissue was then incubated in a PBS solution containing 0.4% Triton-X (Sigma Aldrich, St Louis, MO, USA) and 20% normal goat serum (NGS; Jackson ImmunoResearch Laboratories, Inc., West Grove, PA, USA) for 1 h, and directly transferred to primary antibody guinea pig anti-AgRP (1:40,000; Antibodies Australia, Cat# GPAAGRP.1), diluted in PBS containing 0.4% Triton X-100 and 4% NGS for 17 h. On day three, sections were sequentially incubated in biotinylated goat anti-guinea pig immunoglobulin (IgG; dilution 1:500 in PBS containing 0.4% Triton X-100 and 4% NGS; Vector Laboratories, Burlingame, CA, USA, cat# BA-7000) and Vectastain ABC-elite (dilution 1:500 in PBS; Vector) for 1 hr each with four washes (5 min each) in PBS between incubations. Next, sections were incubated in biotinylated tyramide (BT; dilution 1:250 in PBS containing 3% H_2_O_2_ per mL; Perkin Elmer LAS, Inc., Boston, MA, USA) for 10 min. To visualize AgRP, sections were then incubated in Alexa 488- Streptavidin (dilution 1:100 in PBS; Invitrogen, Carlsbad, CA, USA, cat# S-32354) for 30 min. Following this step, sections were incubated in primary antibody rabbit anti-kisspeptin (1:10,000; Gift from I. Franceschini, cat# 566) diluted in PBS containing 0.4% Triton X-100 and 4% NGS for 17 h. On day four, sections were incubated in biotinylated goat anti-rabbit IgG Alexa 555 (1:100; Invitrogen, cat# A-21428) diluted in PBS, and then washed four times (5 min each) in PBS. Finally, sections were mounted onto Superfrost Plus microscope slides (Fisher Scientific, Waltham, MA, USA), coverslipped using ProLong Gold Antifade Mountant (Fisher Scientific), and stored at 4°C until imaging.

### 4.4. RNAscope In Situ Hybridization for Kisspeptin and Melanocortin Receptors (MC3R, MC4R)

To examine the extent of melanocortin receptor colocalization within ARC kisspeptin neurons of wethers, multi-plex RNAscope was performed for kisspeptin, MC3R, and MC4R. As described above, three sections in the middle ARC were selected, with the right hemisection of the brain used for RNAscope. In situ hybridization was performed based on instructions from Advanced Cell Diagnostics and technical recommendations with minor modifications using the RNAscope Multiplex Fluorescent Reagent Kit v2 (Advanced Cell Diagnostics, Newark, CA, USA; cat# 323100). All incubations between 40 and 60°C were conducted using an ACD HybEZ II Hybridization System with an EZ-Batch Slide System (Advanced Cell Diagnostics; cat# 321710). On day one, hemisections were washed overnight in 0.1 M PBS at 4 °C on a rocking shaker to remove excess cryoprotectant. On day two, sections were submerged in chilled 4% PFA (1 hr at 4 °C), and rinsed four times in 0.1 M PBS (5 min/rinse), followed by an incubation in Hydrogen Peroxide solution (10 min at RT; Advanced Cell Diagnostics, cat# 322335). Hemisections were then incubated with RNAscope Target Retrieval Solution (98 °C for 10 min; Advanced Cell Diagnostics, cat# 322001) and rinsed four times in 0.1M PBS (5min/rinse). Next, sections were mounted onto Superfrost Plus microscope slides (Fisher Scientific), a hydrophobic barrier was created around the tissue using an ImmEdge Pen (Advanced Cell Diagnostics; cat# 310018), and slides were stored overnight at 4 °C. On day three, slides were incubated in increasing concentrations of ethanol (50, 70, 100, and 100%; 5 min each) and allowed to air dry. Sections were then treated with RNAscope^®^ Protease III (30 min at 40 °C; Advanced Cell Diagnostics, cat# 322337), and subsequently incubated with RNAscope target (kisspeptin, Oa-KISS1-C3, cat# 497471-C3; MC3R, Oa-MC3R-C2, cat# 537911-C2; MC4R, Oa-MC4R-C1, cat# 537921-C1) and control probes (positive controls, Oa-UBC-C3, cat#516181-C3; Oa-PPIB-C2, cat# 457031-C2, and Oa-POLR2A, cat# 516171; negative control, 3-plex Negative Control Probe, cat# 320871) for 2 h at 40 °C. Next, slides were washed twice with 1X Wash Buffer (Advanced Cell Diagnostics, cat# 310091; 2 min/rinse at RT) followed by sequential tissue application of 50 µl of the following, each for 30 min at 40 °C with 2 min washes using 1X Wash Buffer between applications: RNAscope Multiplex FL v2 Amp 1 (Advanced Cell Diagnostics, cat# 323101), RNAscope Multiplex FL v2 Amp 2 (Advanced Cell Diagnostics, cat# 323102), and RNAscope Multiplex FL v2 Amp 3 (Advanced Cell Diagnostics, cat# 323103). Following final incubation with Amp 3, slides were rinsed with 1X Wash Buffer twice (2 min/rinse at RT) followed by application of RNAscope Multiplex FL v2 HRP C1 (15 min at 40 °C; Advanced Cell Diagnostics, cat#323104). Slides were then washed with 1X Wash Buffer twice (2 min/rinse at RT), and incubated with 150 µL per slide of Opal 520 (Fisher Scientific; cat#NC1601877) diluted in RNAscope TSA buffer (Advanced Cell Diagnostics, cat# 322809) at a final concentration of 1:1500 for 30 min at 40 °C. Following a rinse with 1X Wash Buffer twice (2 min/rinse at RT), 50 µL of RNAscope^®^ Multiplex FL v2 HRP Blocker (Advanced Cell Diagnostics, cat# 323107) was applied to tissue (15 min at 40 °C). Slides were then rinsed with 1X Wash Buffer twice (2 min/rinse at RT) followed by application of RNAscope Multiplex FL v2 HRP-C2 (15 min at 40 °C; Advanced Cell Diagnostics, cat# 323105). Next, following two rinses with 1X Wash Buffer (2 min/rinse at RT), slides were incubated with 150 µL per slide of Opal 570 (Fisher Scientific; cat# NC601878) diluted in RNAscope TSA buffer (Advanced Cell Diagnostics, cat# 322809) at a final concentration of 1:1500 for 30 min at 40 °C. Subsequently, after two rinses in 1X Wash Buffer (2 min/rinse at RT), 50 µL of RNAscope^®^ Multiplex FL v2 HRP Blocker (Advanced Cell Diagnostics, cat# 323107) was applied to tissue for 15 min at 40 °C, followed by rinsing with 1X Wash Buffer twice (2 min/rinse at RT). Next, RNAscope Multiplex FL v2 HRP-C3 (15 min at 40 °C; Advanced Cell Diagnostics, cat# 323106) was applied to tissue. Slides were then rinsed with 1X Wash Buffer twice (2 min/rinse at RT), and incubated with 150 µL per slide of Opal 690 (Fisher Scientific; cat# NC1605064) in RNAscope TSA buffer (Advanced Cell Diagnostics, cat# 322809) at a final concentration of 1:1500 for 30 min at 40 °C. Next, slides were rinsed with 1X Wash Buffer twice (2 min/rinse at RT), followed by a final application of 50 µL of RNAscope^®^ Multiplex FL v2 HRP Blocker (Advanced Cell Diagnostics, cat# 323107) for 15 min at 40 °C, and two rinses with 1X Wash Buffer (2 min/rinse at RT). Finally, slides were incubated with 50 µL DAPI, coverslipped with ProLong Gold Antifade Mountant (Fisher Scientific, cat# P36930), and stored at 4 °C until image acquisition.

### 4.5. Confocal Analyses

#### 4.5.1. Immunofluorescence

Confocal imaging of immunostained hemisections was conducted using an LSM710 laser-scanning confocal microscope (Zeiss, Thornwood, NY, USA), with a Plan Achromat 40x/1.1 objective. For each section (three sections per wether), z-stacks (1.0-µm optical sections) were captured in the ARC using consistent acquisition settings used for all images. Confocal images were imported into Zen 3.0SR software (Black edition; Carl Zeiss Microscopy GmBH, Jena, Germany), where AgRP close contacts onto kisspeptin neurons were analyzed by a single observer. Based on the approximate size of each kisspeptin neuron, eight to eleven images (1.0- µm optical section) were analyzed per neuron through the z-plane. In each animal, 40–50 kisspeptin cells in which complete cell bodies (with visible nuclei) were imaged in the z-stack were selected for analysis. A close contact was defined as an immunolabeled bouton in direct apposition (no intervening pixels) to a kisspeptin cell body or proximal dendrite [17]. First, the percentage of kisspeptin neurons receiving one or more AgRP-positive close contact was calculated. Next, a subset of kisspeptin neurons (10 cells per animal) that showed at least one putative AgRP close contact was randomly selected in each wether to quantify all putative AgRP-positive inputs onto the cell body or proximal dendrite. Markers placed on putative contacts during analysis ensured that appositions flanking optical sections were not counted twice. In addition, putative terminals were viewed in orthogonal planes (X, Y, and Z), and only those contacting the kisspeptin neuron in all dimensions were quantified.

#### 4.5.2. RNAscope

Hemisections processed for detection of mRNA for kisspeptin, MC3R, and MC4R were analyzed using an LSM 880 laser scanning confocal microscope (Zeiss). For each section (three sections per wether), z-stacks (1.0-µm optical sections) were captured in the ARC using a Plan Achromat 20x/0.8 objective, with consistent acquisition settings used for all images. Confocal images were imported into Zen 3.0 software (Carl Zeiss Microscopy), where the total number of cells expressing kisspeptin, MC3R, or MC4R mRNA were identified by a single observer. Markers placed on cells ensured that the same cell was not counted twice, and cells in which complete cell bodies were visible were included in the analysis, with DAPI used to visualize nuclear area. Images containing marked cells were imported in to FIJI/ImageJ software, where the numbers of cells were quantified. The degree of double or triple labeling was calculated as a percentage of the total number of kisspeptin cells expressing MC3R and/or MC4R, the total number of MC3R cells expressing kisspeptin and/or MC4R mRNA, and the total number of MC4R-expressing cells containing kisspeptin and/or MC3R mRNA.

## Data Availability

The data presented in this study are available on request from the corresponding author. The data are not public due to small samples size.
